# The viable but non-culturable state in pathogenic *Escherichia coli*: A general review

**DOI:** 10.4102/ajlm.v5i1.368

**Published:** 2016-05-04

**Authors:** Jennifer A. Pienaar, Atheesha Singh, Tobias G. Barnard

**Affiliations:** 1 Faculty of Health Sciences, Department of Biomedical Technology, University of Johannesburg, Johannesburg, South Africa; 2 Water and Health Research Centre, Faculty of Health Sciences, University of Johannesburg, Johannesburg, South Africa

## Abstract

**Background:**

The persistence and pathogenicity of pathogenic bacteria are dependent on the ability of the species to survive in adverse conditions. During the infectious process, the organism may need to pass through certain hostile anatomical sites, such as the stomach. Under various environmental stresses, many bacteria enter into the viable but non-culturable (VBNC) state, where they are ‘alive’ or metabolically active, but will not grow on conventional media. *Escherichia coli* bacteria encounter several diverse stress factors during their growth, survival and infection and thus may enter into the VBNC state.

**Objectives:**

This review discusses various general aspects of the VBNC state, the mechanisms and possible public health impact of indicator and pathogenic *E. coli* entering into the VBNC state.

**Method:**

A literature review was conducted to ascertain the possible impact of *E. coli* entering into the VBNC state.

**Results:**

*Escherichia coli* enter into the VBNC state by means of several induction mechanisms. Various authors have found that *E. coli* can be resuscitated post-VBNC. Certain strains of pathogenic *E. coli* are still able to produce toxins in the VBNC state, whilst others are avirulent during the VBNC state but are able to regain virulence after resuscitation.

**Conclusion:**

Pathogenic and indicator *E. coli* entering into the VBNC state could have an adverse effect on public health if conventional detection methods are used, where the number of viable cells could be underestimated and the VBNC cells still produce toxins or could, at any time, be resuscitated and become virulent again.

## Introduction

The survival of a microbial species largely depends upon its ability to subsist in hostile environments.^1,2^ When environmental conditions become unfavourable, bacteria must be able to withstand stress and assume strategies which permit them to endure until suitable conditions for growth are re-established.^1^ Certain bacterial genera achieve this by developing resistant structures, for example, endospores, whereas many others enter a state of very low metabolic activity, usually referred to as the viable but non-culturable (VBNC) state.^1,2^ This suggests that this state is a unique adaptation strategy used by many bacterial species for long-term survival when exposed to hostile environmental stresses.^3,4^ VBNC cells are characterised by culturability loss on growth agar, which hinders their detection by conventional culture-based techniques. This leads to an underestimation of total viable cells in clinical or environmental specimens and could therefore pose a danger to public health.^3^

*Escherichia coli* (*E. coli*) is a genetically diverse species that contains both commensal and pathogenic variants. Strains of *E. coli* and related Gram-negative coliform bacteria predominate amongst the aerobic commensal flora in the gut of humans and animals.^5,6^ *E. coli* is a non-sporulating, facultative anaerobe^7^ that typically colonises the infant gastrointestinal tract within hours of life, from which time on the host and *E. coli* derive mutual benefit.^8^ In the digestive tract, commensal strains are situated in the large intestine, particularly in the caecum and colon.^7^

There are various pathogenic strains of *E. coli* that possess a variety of disease-causing mechanisms such as diverse toxin, secretion, adhesion and siderophore systems, as well as many others.^6^ The benign *E. coli* strains have the potential to acquire genetic elements encoding for virulence factors and could themselves become pathogenic.^6^

Both non-pathogenic and pathogenic species of *E. coli* have been shown to survive sub-lethal environmental stress conditions by entering into the VBNC state.^9,10,11,12,13^ Virulent bacteria, such as pathogenic *E. coli* that are able to enter into a VBNC state, are a major public health concern, because they are able to return to the infectious state after transport in animal hosts.^14^ Reports indicate that many species of pathogenic bacteria survive treatment and persist in pasteurised milk, processed food and drinking water, as well as in the environment.^14^ Water is routinely tested for indicator bacteria (such as *E. coli* and *Enterococcus faecalis*) and if they are not detected at a concentration below the guidelines, the water is considered to be safe for consumption. Thus, in circumstances where there is a chance of VBNC pathogens, additional molecular tests would be required in order to reduce potential outbreaks of infectious disease.^15^ Many studies conducted over the past 30 years have shown that the VBNC state is a significant strategy for bacterial survival.^3^ The presence of cells in the VBNC state in water (and food) have various significant consequences, an example being that *E. coli* cannot be used as an indicator of faecal contamination when the cells are in the VBNC state.^16^

The purpose of this review was to highlight the possible impact on public health of *E. coli* entering into the VBNC state and may be of interest to environmental scientists and medical clinicians. We systematically searched scientific literature databases (Science Direct, Springer, Google Scholar, Wiley, Directory of Open Access Journals, PLoS, NCBI) using keyword combinations of terms such as ‘viable but non-culturable’, ‘*Escherichia coli*’, ‘virulence’, ‘resuscitation’, ‘induction’ and ‘pathogens’. Articles older than 10 years were avoided, when possible, in order to ensure that the latest information on the topic was obtained. All studies involving the VBNC state were included, particularly those relating to *E. coli*.

## Viable but non-culturable state: What exactly does this mean?

The VBNC state is defined as a state of dormancy that certain species of bacteria can enter into when confronted with adverse environmental conditions^15^ such as extreme temperature changes,^2,13,15,17^ starvation,^13,15,17^ high osmotic pressure,^2,13,15,17^ exposure to chlorine,^13,15^ sharp changes in pH,^15^ oxygen availability, heavy metals, or exposure to white light.^15,17^ These conditions could be of a fatal nature if the organism did not enter into a VBNC state.^17^ When bacterial cells are able to grow and form colonies on conventional culture media, they are said to be ‘culturable’, whereas they are ‘viable’ if they are metabolically or physiologically active.^15^ Bacteria in the VBNC state cannot be cultured on routine media; however, they retain metabolic activity, respiration, membrane integrity and slow gene transcription.^17,18^ In spite of their low metabolic rate in this state, after specific resuscitation protocols they may again become culturable.^19^

In an archetypal VBNC response, when the VBNC cells are exposed to environmental stresses a regular decline in colony-forming units is seen. However, total cell counts remain fairly constant. The viable count is very important in determining the VBNC state, as this will show whether the unculturable colonies are dormant but alive, or dead. There are several methods that can demonstrate viability, but all show some aspect of intact bacterial membranes and/or cellular metabolic activity.^17^

## Bacteria known to enter the viable but non-culturable state

To date, the list of bacteria that have been shown to enter the VBNC state constantly increases; some of the notable human pathogens are pathogenic *E. coli*,^13,15,17,19,20^ *Mycobacterium tuberculosis*, *Campylobacter* spp., *Klebsiella pneumoniae*, *Helicobacter pylori*, *Listeria monocytogenes*,^15,17,19,20^ *Pseudomonas aeruginosa*, several *Salmonella* and *Shigella* spp.,^13,15,17,19,20^ *Vibrio cholerae* and *Vibrio parahaemolyticus*.^15,17,19,20^ Universal indicators of faecal contamination, namely, commensal *E. coli* and *E. faecalis*, also enter into the VBNC state.^15^

## Cell changes in the viable but non-culturable state

Bacteria in the VBNC state generally exhibit dwarfing (decrease in cell size),^17,19,21^ acquire a coccal morphology,^21^ show pronounced metabolic changes such as decrease in respiration rates and nutrient transport^17,19^ and macromolecular synthesis.^17,19,21^ Plasmids are retained and adenosine triphosphate levels and membrane potential remain high.^19^ There can be variation in nucleic acid content (particularly RNA) when bacteria enter the VBNC state.^21^

Day and Oliver^22^ found that there were significant changes in membrane fatty acid composition of *Vibrio vulnificus* after incubation at 5 °C in seawater. The results indicated that changes in the fatty acid composition occurred prior to entry into the VBNC state, suggesting that the capability to maintain fluidity of the membrane may be an aspect in this physiological response. Cell death occurred in bacteria where there was inhibition of fatty acid synthesis, indicating that fatty acid synthesis is essential for cells entering the VBNC state.

## Cellular repair mechanisms

Microbial recovery is characterised by the ability to revert to a functionally ‘normal’ state after enduring damage to essential components. This may be achieved by a resuscitation period in a favourable environment.^23^ After exposure to potentially harmful environmental factors, microorganisms may be classified as either ‘dead’ (irreversibly or lethally injured) or ‘alive’. Live cells can be further classified as ‘uninjured’ (normal) or ‘injured’ (stressed, reversibly or sub-lethally injured).^23^ Prolonged exposure of bacterial cells to a principal stress may ultimately lead to irreversible cell death. However, if the injured cells are removed from the environment that is causing the stress, cell recovery may then occur. The ability of the microorganism to once again be culturable on selective media is interpreted as a recovery from the initial injury and points to a repair of the particular metabolic and synthetic functions that were damaged whilst under the particular stress.^24^ Depending on the type and degree of stress, specific biochemical events will be required for repair to occur. The synthesis of DNA, RNA, adenosine triphosphate and proteins, as well as the reorganisation of existing macromolecules, are some of the metabolic processes that occur during repair. Bacteria also amass intracellular compounds that protect membranes and macromolecules from injury.^25^ These repair mechanisms are achieved via signal transduction systems that sense environmental stresses and regulate synchronised expression of various genes involved in mechanisms of cellular defence.^26^

In *E. coli*, trehalose has been recognised as the primary protective osmolyte; trehalose biosynthesis is induced by osmotic shock, extreme heat and cold, desiccation and entry into stationary phase.^25^ Trehalose synthesis is under RNA polymerase, sigma S (RpoS) regulation. As with other polyols, trehalose protects proteins from denaturation by heat and may also protect against pressure.^27^ In enteric bacteria such as *E. coli*, RpoS is the main regulator of the general stress response. Various stress factors affect *rpoS* transcription in different ways with or without the assistance of other cellular regulatory proteins. Under normal circumstances, RpoS levels are relatively low as *rpoS* messenger RNA forms a stable secondary structure, which results in reduced transcription; ClpXP protease also repeatedly degrades the protein.^26^

## Detection of pathogens in the viable but non-culturable state

Because bacteria in the VBNC state are no longer culturable, alternate methods must be employed to demonstrate whether or not these cells are alive.^19^ Generally, these assess viability by testing any of the following criteria: presence of an intact cellular membrane, demonstration of metabolic activity,^4,19,20^ expression of specific genes (e.g. 16S ribosomal RNA) and differential expression of specific proteins (e.g. beta-d-glucuronidase in *E. coli*).^4^ 16S ribosomal RNA has been used in several VBNC studies, because these cells uphold levels of ribosomal RNA as high as normal cells and retain reductase activity (essential for living cells).^4^ Bacteriophages have also been revealed to be useful in detection of VBNC cells, as phage replication properties of live cells can be shown.^28^ Table 1 shows some of the methods employed to detect VBNC bacteria.

**TABLE 1:** Methods of detection of viable but non-culturable bacteria.

Method	Function/Indicator	Potential advantages and disadvantages of methods	Reference
Autoradiography	*De novo* protein synthesis	Advantages: sensitive Disadvantages: radioactivity risk, expensive	20
Fluorescent microscopy	Enzyme activity, membrane integrity, ratio of DNA to protein	Advantages: multiplexing, sensitive, specific, rapid results Disadvantages: autofluorescence, expensive equipment	4, 15, 17, 19, 20
Real-time, reverse transcriptase (RT) polymerase chain reaction (PCR)	Quantitation of 16S ribosomal RNA	Advantages: sensitive, specific, rapid results Disadvantages: expensive equipment	4, 15, 19
Fluorescent *in situ* hybridisation (FISH)	Detection of individual genes	Advantages: rapid results, multiplexing Disadvantages: expensive equipment	4, 15
Bacteriophages	Lytic activity of live cells	Advantages: easy and inexpensive production, specificity, robust Disadvantages: potential inhibition inherent in the bacteria	28
Flow cytometry	Membrane potential, membrane integrity, intracellular enzymatic activity	Advantages: accurate, rapid results, sensitive, multiplexing Disadvantages: expensive equipment, highly skilled operators	17, 29, 30

## *Escherichia coli* in the viable but non-culturable state

Because of both biotic and abiotic ecological factors, aquatic ecosystems such as rivers and oceans represent a hostile environment for allochthonous bacteria such as *E. coli*;^31,32^ thus, these bacteria have developed the VBNC state.^32^ The survival and growth of *E. coli* in foods depends on the interaction between extrinsic or environmental factors (such as pH, temperature and water activity) and intrinsic factors (i.e. those related to food).^26^ The non-culturability linked with the VBNC state poses a potential public health problem, because the methods that are generally employed for detection and counting of *E. coli* depend on culturing.^32^

**FIGURE 1:**
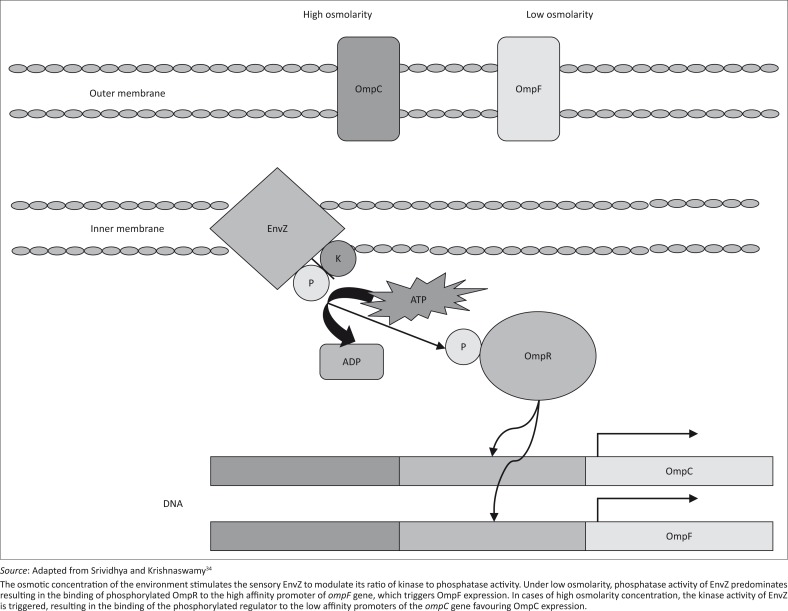
Diagrammatical representation of the EnvZ/OmpR osmoregulatory system in *Escherichia coli*.

Under altered environmental conditions, porins are vital for the survival of bacteria.^33^ The major outer membrane proteins (Omp) in *E. coli* are OmpF and OmpC, whose gene expression is regulated by EnvZ (an osmolarity sensor protein) and OmpR, which responds primarily to changes in osmolarity.^33^ Figure 1 illustrates the EnvZ/OmpR osmoregulatory system in *E. coli*.^34^ A study by Darcan et al.^33^ found that wild-type and porin mutant (OmpC or OmpF deficient) *E. coli* populations entered VBNC under stress conditions of pH, osmolarity and starvation, whereas EnvZ mutants (deficient of EnvZ) were not able to enter a VBNC state. The researchers concluded that because the EnvZ mutant population could not sense the environmental changes, they did not enter into a VBNC state when exposed to the tested adverse conditions. The analysis of the outer membrane subproteomes in VBNC *E. coli* was studied by Muela et al.,^32^ who discovered sets of proteins that were modulated during VBNC induction. Antigen 43 beta-subunit, outer membrane protein TolC and OmpT were modulated by starvation, OmpA and NlpA (lipoprotein 28) by photo-oxidation, and Fiu (catecholate sidephore receptor fiu), FepA (ferrienterobactin receptor), antigen 43 alpha-subunit and adenosine triphosphatase by seawater exposure. Only four identified proteins (Elongation Factor-TU, δ-3 phosphoglycerate dehydrogenase, threonine synthase and enolase) showed an alteration in their expression regardless of the stress factor employed. OmpA maintains the structural integrity of the outer membrane and is an essential protein in *E. coli*; decreases in its expression have been associated with culturability loss in aquatic environments. Elongation factor-TU has an essential role in biosynthesis and becomes membrane bound when *E. coli* is starved of certain nutrients.^32^ Another study^10^ reported that enterohaemorrhagic *E. coli* (EHEC) O157:H7 displayed markedly increased levels of OmpW and a prevalence of Elongation Factor-TU protein after induction into the VBNC state using hydrogen peroxide. In 2008, a study^35^ showed that after passage in food through a mouse, the OmpW stress response of EHEC O157:H7 increased (increase in OmpW expression) when induced into the VBNC state. The authors suggested that the different stress response of OmpW was introduced by means of *in vivo* passage genetic alteration.^35^

Pathogenic EHEC attach and efface intestinal mucosal cells and secrete cytotoxic shiga toxins (Stx) 1 and 2 through the outer membrane.^36^ Consequently, membrane fluidity may play a vital role in the secretion of these toxins. In an attempt to cope with environmental stresses, bacteria induce an alteration in the composition of membrane lipids.^36^ Yuk and Marshall^36^ investigated the relationships between acid resistance and composition of membrane lipids, as well as membrane lipid composition and Stx secretion in three *E. coli* strains: *E. coli* O157:H7; an *rpoS-*deficient mutant of EHEC (EHEC-RM); and non-pathogenic *E. coli* (NPEC). They found that decimal reduction times (D-values) in simulated gastric fluid of cells adapted to acid were higher than those of non-acid-adapted cells, regardless of the strain. In microbiology, the decimal reduction time is used when assessing thermal death time and thermal resistance. It is the exposure time required to cause death in 90% of the initial population under constant temperature and under specified conditions.^37^ Acid adaptation levels decreased *cis*-vaccenic acid (C18:1_7c) and increased palmitic acid (C16:0) in the membrane lipids of all strains. The ratio of *cis*-vaccenic acid to palmitic acid increased at acidic pH, which caused a decrease in the fluidity of the membrane. The greatest Stx concentrations of 2470 ng/mL (EHEC) and 1460 ng/mL (EHEC-RM) were seen in EHEC adapted to pH 8.3 and EHEC-RM adapted to pH 7.3. Furthermore, the ratio of extracellular to intracellular Stx concentration decreased at acidic pH, possibly as a result of the reduction of membrane fluidity. The results may suggest that whilst the *rpoS* gene does not directly affect acid resistance in acid-adapted cells it causes a decrease in membrane fluidity which may, in turn, cause decreased Stx secretion and increase acid resistance.^36^

Despite extensive investigation of VBNC state, relatively little is known about its genetic control.^3^ Nonetheless, two regulators, RpoS and OxyR (DNA-binding transcription dual regulator), appear to be important for induction of the VBNC state.^3,38^ RpoS is a sigma factor essential for general stress response and survival in the stationary phase and has been shown to mediate expression of 10% of the genome in *E. coli* upon exposure to conditions that cause stress.^3,38^ Various studies have suggested RpoS involvement in the survival of *E. coli* in the VBNC state.^18,38,39^ Kusumoto, Asakura and Kawamoto^18^ demonstrated that RpoS mutants (inactivated *rpoS* gene) resulted in faster induction into the VBNC state in *E. coli* and *Salmonella* spp. Boaretti et al.^38^ established that lack of RpoS resulted in diminished ability of *E. coli* cells to remain in a VBNC state for long periods of time, which led to more rapid cell death. After 33 days in an artificial oligotrophic medium incubated at 4 °C, the parental strain of *E. coli* became non-culturable, whereas the *rpoS* mutant lost culturability in only 21 days.

A study conducted in 2005^39^ proposed that reactive oxygen species play a role in the formation of VBNC cells. Desnues et al.^40^ showed that VBNC *E. coli* display reduced superoxide dismutase activity, which resulted in an increase in oxidative damage. Arana et al.^41^ subjected *E. coli to* hydrogen peroxide treatment and showed that some of the cells entered into the VBNC state. Stationary phase *E. coli* were inoculated into filter-sterilised river water samples, then exposed to varying concentrations of hydrogen peroxide for 15 min at 20 °C. The total number of cells (control group), as determined with acridine orange direct count, was higher than the colony-forming unit counts (culturable count) of *E. coli* exposed to hydrogen peroxide. These results suggest that oxidative stress response regulation may be involved in the initiation of the VBNC state. Table 2 summarises some of the proteomic changes seen in *E. coli* that have entered into the VBNC state, as well as which of these are under RpoS and/or OxyR control.

**TABLE 2:** Reported proteomic changes in the viable but non-culturable state in *Escherichia coli*.

Protein/mRNA	Change in the VBNC state	Possible significance	Reference
Elongation factor-TU	Maintained expression	Suggests maintenance of protein synthesis.	10, 32
Diaminopimelic acid (DAP)-DAP muropeptides	Increase in cross-linking	May be an expedient for producing cross-linkage of peptidoglycan in conditions such as VBNC where there is a shortage of pentapeptide donors.	42
Muropeptides (containing tripeptide)	Increase	Connected to shape transition.	42
Glycan strands	Decrease in length	Connected to shape transition.	42
Penicillin-binding proteins	No longer present	Block or decrease in peptidoglycan assembly and growth.	42
OmpW	Increased post *in vivo* passage	Sensitisation to stress (increase the stress response).	10, 35
*rpoS* (mRNA levels were measured)	Persisted	Implies the involvement of the rpoS gene (and thus protein) in the persistence of *E. coli* in the VBNC state.	38
*gapA* (mRNA levels were measured)	Persisted/increased	Maintenance of glycolysis.	43
16S RNA (mRNA levels were measured)	Persisted/increased	Maintenance of ribosomal functioning.	43
Enolase	Persisted	Maintenance of glycolysis.	32
δ-3-phosphoglycerate dehydrogenase	Persisted	Maintenance of amino acid synthesis.	32
Threonine synthase	Persisted	Maintenance of amino acid synthesis.	32

VBNC, viable but non-culturable; mRNA, messenger RNA.

## Induction into and *in vitro* resuscitation of *E. coli* in the viable but non-culturable state

Numerous environmental and chemical factors have been reported to induce the VBNC state.^19^ Entry into the VBNC state is usually as a result of a natural stress, for example starvation; however, cells may also enter into this state during processes that are normally thought of as bactericidal, such as wastewater chlorination and pasteurisation of milk.^17^ This has implications for public health, as some of the established methods for food and water sanitation may, in fact, induce the VBNC state. Table 3 summarises a number of methods that have been reported to induce the VBNC state in *E. coli*.

**TABLE 3:** Inducers of viable but non-culturable state in *Escherichia coli*.

Inducing factor	Reference
Starvation	9, 11, 12, 16, 32, 33
Suboptimal temperature	12, 37
Chlorination	11, 16
Osmotic stress	43, 44
High pressure CO_2_ (HPCD)	13
Oxidative stress	10, 35, 40
Visible radiation	9, 32
Sunlight	45
pH variation	33

It is important to note that the VBNC phenotype is reversible; that is, bacteria that enter this state may again become culturable.^3^ The transition of bacterial cells from the VBNC state back to a culturable state is known as resuscitation.^46^ Various authors theorise that the VBNC state forms part of the bacterial life cycle and hence constitutes a survival strategy in the face of unfavourable conditions. Conversely, other studies have reported that non-culturable bacteria cannot be resuscitated.^1^ The underlying idea is that the VBNC phenotype is only of interest if cells in this state can resuscitate back to a state of culturability.^2^ Although rich media was the first stimulus found to induce resuscitation, subsequent studies have identified a variety of stimuli that can trigger resuscitation.^3^ The following are some of the mechanisms that have been used to resuscitate bacterial cells from a VBNC state: use of host cells (e.g. embryonated chicken eggs); presence of specific compounds, such as amino acids, resuscitation promotion factor and autoinducers;^47^ and the removal of environmental stress (e.g. addition of nutrients to starved cells, return to darkness, change to a suitable temperature).^3^

It is important to note that for VBNC detection and resuscitation, a rich medium should generally be used, since some cells that may have been injured (but have not become VBNC) during exposure to the VBNC-inducing stress might not be capable of growing on differential or selective media containing antibiotics and/or other restrictive compounds.^3^ These injured cells have a greater sensitivity to components of the growth medium that are not customarily inhibitory, but they are not considered VBNC cells since they can be cultured on non-selective media.^3^

Ohtomo and Saito^48^ demonstrated that resuscitation from the VBNC state to the culturable state occurs in *E. coli* with the removal of environmental stress. Exposure of these cells to high saline stress caused a significant decrease in the number of colony-forming units, but when the culture was relieved of the stressful condition the number of colony-forming units returned, within two hours, to the same level as before the stress.

In 2011, Pinto et al.^46^ tested the ability of VBNC *E. coli* to resuscitate under a minimal medium supplemented with different amino acids, discovering that a combination of glutamine, threonine, leucine and methionine would be adequate to trigger resuscitation of one of the tested strains (Eco3). It was proposed that in order to initiate resuscitation, these amino acids may bind to receptors on the cell surface or be transported into the cells. Additionally, they found that increased resuscitation was observed at 37 °C as opposed to 25 °C.

**FIGURE 2:**
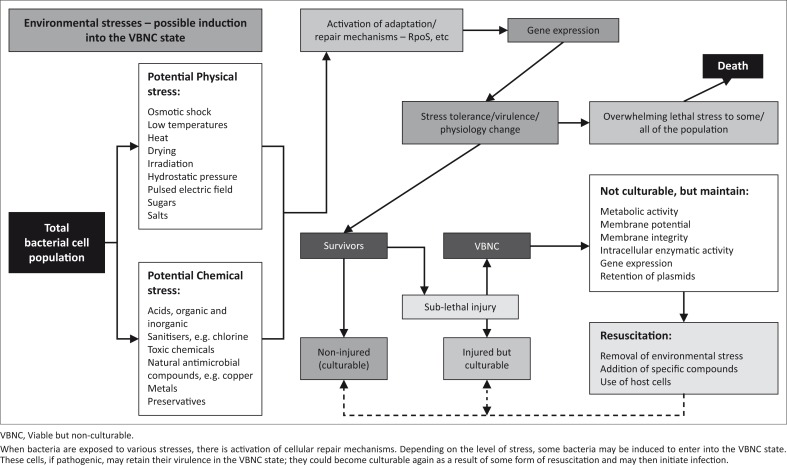
The viable but non-culturable state.^19, 23, 24, 25, 47^

Bacteria communicate with each other by means of a process known as quorum sensing and can modulate population density-dependent behaviour such as biofilm formation and pathogenicity. Population size estimation is accomplished by producing and responding to certain chemical signalling molecules.^49^ It has been shown that *E. coli* use the S-Ribosylhomocysteinase (luxS)/autoinducer (AI)-2 quorum sensing system for population communication and were found to produce at least two autoinducers, AI-2 and AI-3.^50^ One study^47^ showed that EHEC O157:H7 can be resuscitated using its ALS, such as AI-2, that are produced during biofilm formation. That fact that AIs support resuscitation of bacteria in the VBNC state has a significant implications, in that commensal *E. coli* in the intestine can produce AIs similar to those produced by EHEC O157:H7.^47^ Thus, the human intestine could prove a suitable environment for these pathogenic VBNC cells to undergo restoration of culturability. Figure 2 summarises the VBNC state and the connection to repair mechanisms, induction and resuscitation.

## Potential virulence of *E. coli* in the viable but non-culturable state

For those species of bacteria that cause human infections, the underestimation or even non-detection of viable cells in quality control samples from clinical samples, water distribution systems and the food industry, may present a grave public health risk.^3^ Quite a number of cases of bacterial infection are not linked with the isolation of the causative agent.^51^ It has been theorised that this may be as a result of a viral cause, low bacterial concentrations or non-culturability of the bacteria. During infection, these pathogens may reach sites in the body where they are exposed to molecules that hinder their growth (e.g. exogenous antibacterial drugs, substances made by the host, or the host’s resident bacterial population). Stress conditions found *in vivo* may induce the bacteria into a VBNC state.^51^ Bacterial populations in the VBNC state have consequences in their own right, because they maintain activity and thus participate in the functioning of the ecosystems, in energy production and in the carbon cycle. Furthermore, pathogenic bacteria in the VBNC state are still able to produce toxins, thereby having a negative effect on their host.^1^

Pathogenic *E. coli* are classified into groups based on their mechanisms of pathogenicity, clinical syndromes, virulence factors and distinctive O:H serotypes. The overall pathogenesis of these strains consist of mucosal site adhesion/colonisation, host defence evasion, multiplication and host damage.^52^ The groups include enterotoxigenic *E. coli*, enteropathogenic *E. coli*, enteroinvasive *E. coli*, enteroaggregative *E. coli* and enterohaemorrhagic *E. coli*.^26^ A summary of the individual virulence mechanisms of these strains is outlined in Table 4.

**TABLE 4:** Mechanisms of virulence in pathogenic *Escherichia coli*.

Pathogenic strain	Primary virulence mechanisms	Reference
Enterotoxigenic *E. coli*	*Adhesion*: caused by fibrillar colonisation factors (colonisation factor antigen [CFA]/I, CFA/II, or CFA/IV). *Toxin production*: Heat-labile enterotoxins (LTs) and/or heat-stable enterotoxins (STs) are produced. LTs activate the main chloride channels of epithelial cells, and STs activate guanylate cyclase activity. The activity of either toxin results in influx of water into the intestines.	6, 52
Enteropathogenic *E. coli*	*Adhesion and mucosal damage*: an attaching and effacing (A/E) histopathological lesion is induced by a protein, intimin, and various other chaperones and effector proteins (such as a translocated intimin receptor) which are coded for by genes on a pathogenicity island called the locus of enterocyte effacement. *Immune suppression*: lymphostatin inhibits lymphocyte activation. *Toxin production*: some enteropathogenic E. coli strains produce an enterotoxin called EspC causing direct cytotoxic effects in epithelial cells.	6, 52
Enteroinvasive *E. coli*	*Intestinal invasion and damage*: invasion and dissemination into epithelial cells are as a result of gene products present on a large virulence plasmid. After epithelial cell penetration, there is endocytic vacuole lysis, intracellular multiplication and finally spread to neighbouring cells, resulting in tissue destruction and inflammation.	6, 52
Enterohaemorrhagic *E. coli*	*Adhesion and mucosal damage*: enterohaemorrhagic E. coli also produce an attaching and effacing (A/E) histopathological lesion due to the secretion system of the locus of enterocyte effacement pathogenicity island. *Toxin production*: potent shiga toxins (Stx1 and Stx2) are produced. Shiga toxin is composed of two major subunits, A and B. The B subunit binds to globotriaosylceramide-3, found on many different cell types, and causes damage via a combination of direct toxicity and cytokine induction. The A subunit cleaves ribosomal RNA which results in protein synthesis inhibition and induction of apoptosis. Shiga toxins also mediate local damage in the colon, which results in intestinal perforation, haemorrhagic colitis, bloody diarrhoea and necrosis.	6, 52, 53
Enteroaggregative *E. coli*	*Adhesion*: adherence in an aggregative, stacked-brick-type pattern to the intestinal mucosa is thought to occur via one of several different aggregative adherence fimbriae. *Toxin production*: Several toxins have been described. Shigella enterotoxin 1 is one of the toxins produced, but the mode of action is not yet understood.	6, 52, 54

Viable but non-culturable pathogenic *E. coli* have been implicated in a variety of diseases. Pathogenic bacteria, whilst in the VBNC state, can be avirulent. The problem arises when some of the organisms can regain their virulence after resuscitation into culturable cells under suitable conditions.^3^ Table 5 summarises the pathogenic *E. coli* that have been shown to enter the VBNC state.

**TABLE 5:** Pathogenic *Escherichia coli* known to enter the viable but non-culturable state.

Pathogenic strain	Mechanism(s) of induction into the VBNC state	Reference
Enterotoxigenic *E. coli*	Salt water; sunlight; starvation	44, 45, 55
Enterohaemorrhagic E. coli	Oxidative stress (H_2_O_2_); High Pressure CO_2_; salt water; chlorination; starvation	10, 11, 13, 35, 43, 55
Enteropathogenic *E. coli*	Starvation	55
Enteroaggregative *E. coli*	Starvation and/or copper ion treatment; chlorination	56

VBNC, viable but non-culturable.

Liu et al.^11^ showed that EHEC O157:H7, when exposed to various environmental stresses, entered into the VBNC state and was still able to produce potent shiga toxins (both Stx1 and Stx2), which are responsible for the chief symptoms of haemolytic uremic syndrome and haemorrhagic colitis. In 1998, an EHEC O157:H7 outbreak in salted salmon roe occurred in Japan. It was found that patient samples containing O157:H7 would not grow on culture media after incubation in 13% NaCl, but after being resuscitated in yeast extract broth, 90% of the cells were shown to be viable. These findings suggested that almost all cells are capable of entering into the VBNC state when exposed to salt water.^41^

Zhao et al.^13^ induced *E. coli* O157:H7 into the VBNC state by exposure to high-pressure CO_2_ treatment (one of the non-thermal techniques used for pasteurisation). They were able then to resuscitate the organism using tryptic soy broth at 37 °C. Their results demonstrated that high-pressure CO_2_ treatment could induce *E. coli* into the VBNC state, which has future public health implications since this technique is operational and soon to be employed for pasteurisation of liquid foods (such as fruit juice and milk) on a commercial scale.^57^ They concluded that all products treated by high-pressure CO_2_ treatment should be checked for VBNC populations of bacteria using molecular-based methods in order to ensure the safety of the product.

Waste water forms the chief reservoir of human enteric bacteria such as *E. coli*. Enteric bacteria are potential sources of disease; thus, it is imperative to properly disinfect waste water before it reaches the intakes for water treatment plants. Chlorine, in the form of hypochlorous acid, is generally used to this end as it is an exceptionally powerful antibactericidal agent.^16^ In 2005, a study^16^ found that when *E. coli* K12 and *S. typhimurium* cells were exposed to a mixture of free and combined chlorine (as is used in waste water disinfection), a small proportion of the cells survived in the VBNC state. The researchers were not successful in resuscitating the cells, but concluded that the presence of the non-culturable cells still posed a possible threat to public health as evidence from other studies indicate that the cells of *E. coli* and *S. typhimurium* are capable of resuscitation. Another study^45^ tested the effects of sunlight and seawater on *E. coli* H10407 and found that the bacteria entered into a VBNC state after exposure to both solar irradiation and seawater. The researchers established that *E. coli* retained its pathogenicity in the VBNC state, as enterotoxins were still produced. Lothigius et al.^44^ determined that enterotoxigenic *E. coli* may still be metabolically active and viable after incubation in both fresh- and salt water for extended periods of time because of the VBNC state. It was found that the cell wall remained intact and there was expression of both metabolic and virulence genes after three months of incubation in water.^43^

Another significant health implication of the VBNC state in pathogenic *E. coli* is possible antibiotic resistance in such cells.^3,4^ VBNC cells have a low metabolic rate and thus antibiotics targeting components or activities in active cells may prove less effective in quiescent cells.^3,4^ The VBNC bacterial populations may develop resistance to antibiotics, then resuscitate and initiate infection.^19^ An additional possibility is that the antibiotic acts an inducer for the VBNC state, as suggested by a study by Mason et al.,^58^ where *E. coli* was exposed to 10 or 100 times the minimum inhibitory concentration of ciprofloxacin. The results showed that the colony-forming units decreased over time; however, there was no decrease in total cell numbers as seen by means of flow cytometry and light microscopy.^58^

### Conclusion

VBNC pathogenic *E. coli* pose a public health risk, particularly those that may be present in water or food, because they still display metabolic activity, but cannot be detected via standard laboratory techniques, such as culturability.^12^ The existence of the VBNC state raises some serious questions about quality procedures that were previously thought to be relatively fool-proof, such as antibiotics testing, the sterility of medicinal drugs and the interpretation of routine food and water testing. It masks the actual number of viable cells that may, at any time, be resuscitated, emphasising the need to be aware that this state exists and take it into account when doing quality testing.^1^
